# Monoclonal Gammopathy of Renal Significance with Deposits of Peculiar Morphology and Injuries of Secondary Thrombotic Microangiopathy: A Case Report and Review of the Literature

**DOI:** 10.1155/2020/6679857

**Published:** 2020-12-08

**Authors:** José C. De La Flor, Marina Alonso, Edna Sandoval, Alexander Marschall, Miguel Rodeles

**Affiliations:** ^1^Department of Nephrology, Hospital Central Defense Gomez Ulla, Madrid, Spain; ^2^Department of Anatomic Pathology, Hospital 12 de Octubre, Madrid, Spain; ^3^Department of Hematology, Hospital Central Defense Gomez Ulla, Madrid, Spain; ^4^Department of Cardiology, Hospital Central Defense Gomez Ulla, Madrid, Spain

## Abstract

We present the case of an 82-year-old woman diagnosed with monoclonal gammopathy of renal significance (MGRS) with the presence of different and peculiar kidney lesions, who began treatment with bortezomib and dexamethasone, presenting during her evolution a relapse. Although the bone marrow biopsy in this case showed plasma cells as pathologic clone and there was also a reduction after chemotherapeutic treatment, rituximab was proposed as a second line. We suspected that the relapse was possibly due to another precursor as B-cell or lymphoplasmacytic cell clone. We review the literature and suggest that the treatment for MGRS should be patient-tailored, preferably by consulting a multidisciplinary team. Future research is needed to better understand the disease course and establish the efficacy and safety of the therapeutic approach for the relapse of MGRS.

## 1. Introduction

The monoclonal gammopathy of renal significance (MGRS) is pathogenically characterized by the proliferation of B lymphocyte clones or plasma cell clones that synthesize and secrete a monoclonal immunoglobulin (Ig) or its components (light or heavy chains), with the ability to cause damage at the tubular, glomerular, vascular, and interstitial compartments through direct (deposition) or indirect (alterations of the alternative complement pathway) mechanisms, constituting a heterogeneous group of entities. It is unusual to find more than two forms of monoclonal immunoglobulin lesions as well as different injury mechanisms in the same biopsy, as in the case of our patient.

Proliferative glomerulonephritis with monoclonal immunoglobulin deposits (PGNMID) is a form of MGRS-associated lesions where M-protein is deposited in the glomerulus. According to the classification scheme proposed by the International Kidney and Monoclonal Gammopathy Research Group (IKMG) [1], it belongs to the lesions caused by nonorganized monoclonal immunoglobulin deposits, often leading to chronic or end-stage kidney disease (ESKD) [2, 3].

On the other hand, thrombotic microangiopathy (TMA) is a syndrome physiologically characterized by endothelial injury. TMA may be hereditary or acquired, including a variety of disorders, such as complement-mediated TMA (also known as atypical hemolytic uremic syndrome (aHUS)) and other associated conditions [4]. TMA, according to a consensus report of the IKMG in 2019 [5], belongs to the lesions without deposits. Its pathophysiology is not fully understood, but it might be related to the monoclonal Ig acting as an autoantibody against a complement regulatory protein, which could trigger the development of aHUS and other forms of TMA [6].

The optimal treatment for most subtypes of MGRS is not known, but there is recent consensus among experts that treatment should be specific for the underlying clone [7]. This is probably due to the low detection rate of circulating paraproteins and pathogenic clones in PGNMID, highlighting the need for better diagnostic tools [8]. We report a case of MGRS with lesions of PGNMID, glomerular TMA, and deposits of peculiar morphology with torpid evolution despite chemotherapeutic treatment adapted to the nature of the cell clone.

## 2. Case Presentation

Our patient was an 82-year-old female with a past medical history of arterial hypertension (AHT) and dyslipidemia. Her medication included angiotensin II receptor blockers (ARBs) and statin. The patient presented with asthenia, fatigue, and uncontrolled AHT for a one-month duration. On admission, physical examination revealed a blood pressure of 250/130 mmHg, a respiratory rate of 24 breaths/min, an oxygen saturation of 90% while breathing room air, pulmonary rales, and peripheral edema. Furthermore, the patient presented with fever and low level of consciousness. Her laboratory values indicated a creatinine (Cr) level of 1.93 mg/dl, hypoalbuminemia without hypercholesterolemia, a massive proteinuria of 4.9 g/gCr, and blood smear with schistocytes <1%. Other laboratory test results are shown in Table 1.

**Figure 1:**
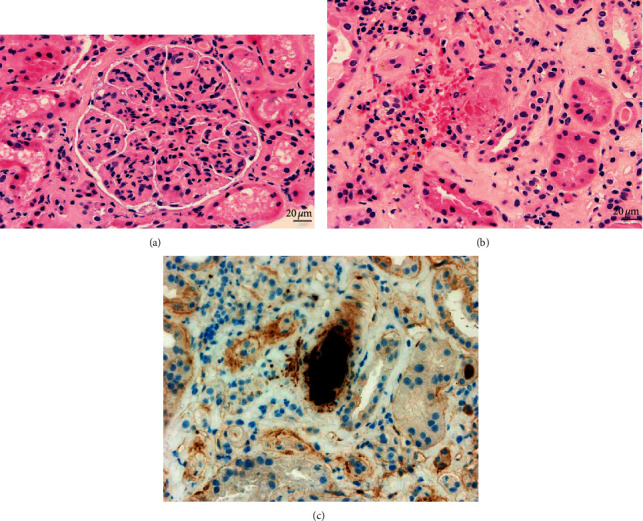
All the glomeruli displayed membranoproliferative features, with increased cellularity and a lobular pattern (a) (H-E, 400x). A single arteriole showed a luminal thrombus, composed of fibrin and platelets, together with blood extravasation and karyorrhectic debris (b) (H-E, 400x). CD61 immunostaining was performed (400x), which demonstrated platelet content (c).

**Figure 2:**
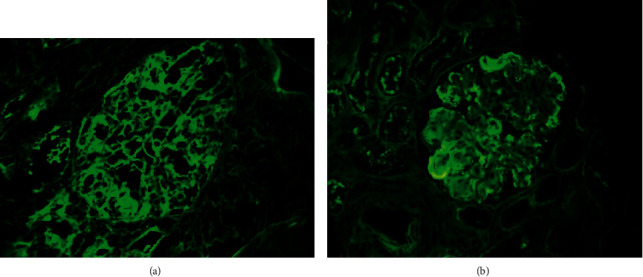
Direct immunofluorescence on fresh frozen tissue was performed; no evidence of deposits was observed (a) (IgG, 400x). Direct immunofluorescence on paraffin-embedded, pronase-digested sections was carried out; in contrast to routine IF, there were deposits of IgG and kappa light chains in the capillary walls (b) (kappa, 400x).

**Figure 3:**
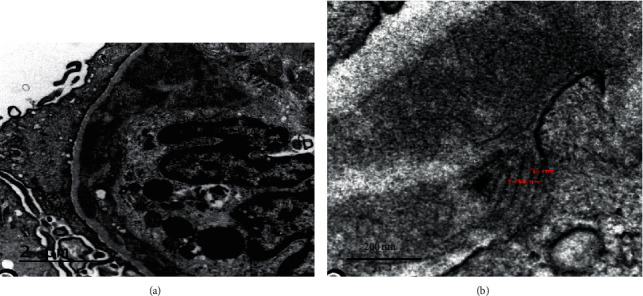
Electron microscopy showed subendothelial and mesangial deposits (a) (10000x); for the most part, these were nonorganized; however, there were some fibrillary deposits with parallel arrangement measuring approximately 10–15 nm (b) (30000x).

Thus, a kidney biopsy was performed; 14 glomeruli could be analyzed. Three of the glomeruli were globally sclerotic. Under light microscopy, all of the remaining 11 glomeruli showed mesangial proliferation and endocapillary hypercellularity without crescent formation. 60% of the glomeruli had mesangial expansion and basement membrane duplication (Jones methenamine silver stain). Congo red and thioflavin staining were negative. One glomerulus showed mesangiolysis and a thrombus at the vascular pole (silver methenamine-stained section) with capillary wall remodeling, multiple layering, and double-contour formation in the section stained with periodic acid-Schiff- (PAS-) positive. The arteries and arterioles showed intimal fibrosis that decreased the light by approximately 25%. In only one of the arterioles, a luminal thrombus with phenomena of hematic extravasation and fragmentation of red blood cells could be seen. Immunohistochemical staining for CD61 was positive in the arteriole described histologically, as well as in some of the glomeruli. There were mild tubular atrophy and interstitial fibrosis as estimated at 15–20% (as shown in Figure 1). Routine frozen tissue immunofluorescence (IF-F) showed four glomeruli with unspecific deposits of C1q and IgM, without the presence of deposits for the rest of the antisera used (IgA, IgG, C4, fibrinogen, kappa, and lambda). On the other hand, the IF on pronase-digested paraffin-embedded (IF-P) sections showed subendothelial deposits of IgG and kappa light chain (++/+++) with negative staining for C3 and lambda light chain (as shown in Figure 2). There was no light chain staining in IF-F nor in IF-P in the tubule and renal interstitium. Detection of IgG subclasses was not performed. Electron microscopy (EM) showed electron-dense nonorganized subendothelial deposits; on this amorphous background, there were some fibrils with parallel arrangement and a diameter ranging from 10 to 15 nm. These fibrils did not correspond to typical amyloid deposits, nor could they be identified as the ones seen in fibrillary glomerulopathy (FGN). The immunohistochemistry technique for DNAJB9 was negative, which ruled out FG (as shown in Figure 3).

Bone marrow (BM) biopsy revealed <3% plasma cells, with negative Congo red staining. The immunophenotypic study showed that 2.1% of the total cellularity were plasma cells and, of these, 97% had an abnormal phenotype with monoclonality for the kappa light chain. IdentiClone assay was not performed. A PET/CT (positron emission tomography-computed tomography) scan revealed the absence of data on lymphoproliferative syndrome, solitary plasmacytoma, and other extramedullary affectations.

Given the diagnosis of MGRS with lesions of PGNMID (IgG-kappa), glomerular TMA, and light-chain deposits of peculiar morphology (DNAJB9 negative) seen in EM, treatment with cycles of bortezomib and dexamethasone (BordD) chemotherapy was initiated and well tolerated. After receiving three cycles, a partial renal response (renal function improved with serum creatinine decreasing to 1 mg/dL and proteinuria >50% to 1.2 g protein/day) and a partial hematological response (reduction >50% of kappa light chain) were achieved. Nevertheless, after the sixth cycle, the patient presented a worsening of kidney function with creatinine increasing to 2.4 mg/dl, proteinuria to 2.7 g/day, and kappa light chain (26.6 mg/d) in an immunophenotypic study that showed a slight decrease in abnormal cellularity (2% of the total cellularity were plasma cells and, of these, 75% had an abnormal phenotype of the kappa light chain).

For this reason, and given the evidence of relapse, different therapeutic options were evaluated (according to the first response, toxicity, general status, and renal function). Treatment with intravenous rituximab (two infusions, 1000 mg each, administered 14 days apart) and prednisone (0.8 mg/kg/day) tapered to 10 mg/d over 3–6 weeks followed by a gradual discontinuation after 3 months, was initiated. Four months later, our patient presented improvement in renal function with a Cr of 1.7 mg/dl and a proteinuria of 1.43 g/day. The following controls were delayed due to the onset of the COVID-19 pandemic and the patient died 3 months later of COVID-19 pneumonia.

## 3. Discussion

The spectrum of renal involvement in MGRS is wide. Most of the cases are due to M-protein deposits, while in other occasions it occurs indirectly through the dysregulation of the alternative complement pathway that leads to C3 glomerulopathy (GNC3) or more rarely to an aHUS [1, 5]. The most accepted classification for renal involvement by deposits is based on the configuration of its structure, dividing the disorder into organized and unorganized, whose ultrastructural measurement is the key to guide the diagnosis and to be able to discern the type of fiber or immunoglobulin deposited [5]. Although in clinical practice it is not uncommon for more than one entity to show an overlap of the damage mechanisms in the MGRS biopsy [9], it is rare to have more than three concurrent monoclonal immunoglobulin-related lesions of different mechanisms of injury in the kidney, which makes it more difficult to classify it [10].

Klomjit et al. [11] recently published the first study to evaluate the rate and clinical predictors of finding an MGRS lesion in a kidney biopsy specimen. They identified 6300 patients who had monoclonal gammopathy (MG) from Mayo Clinic medical records from 2013 to 2018. 160 (2.5%) had undergone a kidney biopsy. Of the 160 patients, 64 (40%) had an MGRS lesion; amyloid light-chain amyloidosis was the most common finding (*n* = 28, 43.8%); PGNMID was the second most common lesion (*n* = 12, 18.8%), followed by light-chain proximal tubulopathy (LCPT) (*n* = 6, 9.4%), light-chain deposition disease (*n* = 5, 7.8%), and type I cryoglobulinemic GN (*n* = 5, 7.8%). 96 patients (60%) had a kidney lesion that was unrelated to the monoclonal protein (non-MGRS group). The authors identified the following clinical predictors for the increase of odds of finding an MGRS lesion: proteinuria ≥1.6 g/d, hematuria, low serum C3 levels, abnormal FLC, and abnormal bone marrow biopsy specimen. Our case presented 4 of the 5 parameters described according to the study of the Mayo Clinic group.

In our case, the renal biopsy showed an MPGN pattern of glomerular injury by light microscopy. In routine IF, it presented a false-negative staining for monoclonal immunoglobulins, which was unmasked by performing IF-P. These paraffin rescue techniques are used when a valid kidney tissue is not available in IF-F. It has a high probability of success for the immunohistochemical detection of light chains [12]. Furthermore, this technique unmasks glomerular monoclonal deposits, and it helped us to establish the diagnosis of PGNMID IgG-kappa. Larsen et al. [13] reported twenty-six patients with a known monoclonal gammopathy who had a kidney biopsy showing C3 only or glomerulonephritis (GN) with negative glomerular staining for IgG, IgM, IgA, kappa, lambda, and C3 by routine IF. However, 21 samples had a residual paraffin tissue to undergo paraffin IF, including 11 cases of C3 GN, 6 dense deposit disease (DDD), and 4 unclassified MPGN/cryoglobulinemic GN cases. Paraffin IF identified 7 (33%) cases (4 of 11 C3 GN cases and 3 of the 4 unclassified MPGN/cryoglobulinemic GN cases) with positive staining for Ig. The authors strongly recommend performing paraffin IF in all patients with clinical evidence of MG in whom kidney biopsy shows C3 GN or MPGN with negative IF findings and when routine IF does not match either the clinical scenario or electron microscopic findings. Masked deposits are a rare phenomenon, and paraffin IF is certainly not warranted in most cases.

The lesions of glomerular TMA associated with MG were also observed in our patient, without presence in the clinical setting of microangiopathic hemolytic anemia (MAHA) and thrombocytopenia, and with normal levels of factor H autoantibody, the diagnosis of aHUS (complement-mediated TMA) was ruled out. Ravindran et al. [6] observed a high prevalence of MG in complement-mediated glomerulonephritis (C3 GN). Monoclonal Ig can cause dysregulation of the alternative pathway of complement by multiple mechanisms. One of these mechanisms is to act as an autoantibody to the complement factor H and interfering with its complement regulating function, and the other is its function as an autoantibody to the complement factor B that is a component of C3 convertase [14, 15]. The authors hypothesized that monoclonal Ig may facilitate the development of TMA directly or indirectly. The M-protein may injure the endothelial cells directly or interfere with the fibrin structure or indirectly via functional inhibition of proteins that regulate thrombosis. Finally, they conclude that all patients with TMA should undergo evaluation for MG. In our patient, the pathophysiological explanation of this disorder is not clear because TMA was not associated with C3 GN. Moreover, the other lesions in this category are glomerular microangiopathy associated with polyneuropathy, organomegaly, endocrinopathy, monoclonal gammopathy, and skin changes (POEMS) syndrome [16, 17]. In these cases, the lesion is a subacute to chronic glomerular thrombotic microangiopathy and is not associated with MAHA. Our patient had a similar injury, but she did not meet the criteria for this syndrome.

The most striking finding was the presence of electron-dense deposits with fibrils of 10–15 nm in the EM. These images are not typically observed in FGN or amyloidosis (fibrils < 20 nm with random orientation in mesangial cells and capillaries). Therefore, these images could correspond to a special way of crystallizing light-chain deposits or are unique features of this biopsy, demonstrating the unusual appearances that abnormal light chains can display. The fibrillar deposits are easy to confuse with each other since we only have the diameter, structure, and organization to differentiate them in EM. For this reason, it is necessary to perform amyloid stains and DNJB9 immunostaining for their better classification. In our case, the immunohistochemistry technique for DNAJB9 was negative. This is in line with a recent study published by Said et al. [18] although monotypic FGN is currently classified as a MGRS. The authors of this study performed IF-P on 35 cases of FGN that showed apparent light-chain restriction on IF-F and correlated the IF-P findings, IgG subtype staining, and SPEP/SIF. Only one of 11 cases had a detectable circulating monoclonal protein on SPEP/SIF. Said et al. conclude that DNAJB9-positive monotypic FGN (confirmed by IF-P and IgG subclass staining) is very rare and is not associated with MGRS in most patients.

MGRS treatment strategies are based on chemotherapy that must be adapted to the nature of the cell clone, both lymphocytic or plasma, renal function, and the presence or absence of extrarenal involvement. The evaluation of hematological response is crucial because the reduction of monoclonal Ig components is associated with the best renal outcomes. However, renal response is not contingent on the resolution of the M-protein in PGNMID [19]. Recently, Van Kruijsdijk et al. [20] reported 2 cases of PGNMID and raised the question if clone-directed therapy is always necessary, given the probability that the M-protein can disappear spontaneously without treatment, in cases when the quantification is only 0.4%. The authors detect incongruities probably because of a small sample size and the risk for confounding by indications that can be made with the results of 65 patients with PGNMID of three observational studies [2, 8, 19]. 73% of the patients who received clone-directed therapy had complete or partial renal response, but also 54% of the patients who received no clone-directed therapy (steroids, mycophenolate mofetil, and/or cyclosporine) and 29% of the patients who received no treatment or renin-angiotensin system blockade alone achieved complete or partial renal response.

In our case, the flow cytometric immunophenotyping of bone marrow showed the presence of plasma cells with abnormal kappa. Therefore, treatment with cycles of BordD chemotherapy was initiated, with good outcomes at the beginning. However, at the end of the sixth cycle, both renal and hematological relapse was evident, despite observing a 26.8% reduction in plasma cells with an abnormal phenotype for kappa. Given the heterogeneity of multiple kidney lesions associated with MGRS, and even more so in cases of relapse, to date, there is little experience regarding the optimal treatment of these complex forms [8, 19]. Different regimens including cyclophosphamide, bortezomib, melphalan, lenalidomide, rituximab, and daratumumab have been used according to the degree of glomerular filtration and proteinuria, with limited results [10]. In our case, rituximab treatment was proposed as a second line. Rituximab is a treatment option for cases of MGRS associated with IgM lambda deposits with identification of the B lymphocyte clone [2]. Despite the fact that the bone marrow biopsy showed plasma cells as pathologic clone and there was also a reduction after chemotherapeutic treatment, we suspected that the relapse of the disease occurred because the detected M-protein could not have been properly produced by this clone, if by another precursor as the B-lymphocyte clone.

New diagnostic tools need to be investigated in cases where the M-protein is little or not detected since clonal identification is even more difficult [7]. Despite the initial improvement with rituximab, we were unable to monitor the response to our treatment because the patient died of COVID-19 at the beginning of the pandemic in Spain.

In conclusion, we recommend that the treatment for MGRS should be patient-tailored, preferably by consulting a multidisciplinary team. Future larger, prospective, and multicenter studies on pathophysiology are needed to better understand the disease course and to establish the efficacy and safety of therapy in the relapse of MGRS.

## Figures and Tables

**Table 1:** Laboratory findings on admission.

		Reference range/unit
WBC	3978	U/L
Hemoglobin (Hb)	11.3	12–16 g/dL
Platelet count (PLt)	168	10^3^/*μ*L
Reticulocyte count	2.77	2–4%
Haptoglobin	93	30–200 mg/dL
Lactate dehydrogenase (LDH)	385	135–214 IU/L
ADAMTS 13 activity	15	<10%
Coombs test	Negative	NA
Total bilirubin	0.3	0.1–1 mg/dL
Total protein (TP)	5.9	6.4–8.7 g/dL
Serum albumin (Alb)	2.6	3–5.5 g/dL
GOT	29	5–32 IU/L
GPT	21	5–33 IU/L
Urea	83	17–60 mg/dL
Creatinine	1.93	0.6–1.2 mg/dL
Na	141	135–145 mmol/L
K	4.2	3.5–5.5 mmol/L
Cl	101	95–110 mmol/L
CRP	2.51	0.1–0.5 mg/dL
HbsAg	Negative	NA
HCV-Ab	Negative	NA
HIV	Negative	NA
CH50	45	40–90 U/ml
CFH	250	225–760 *μ*g/mL
Autoantibodies CFH	Negative	<18 AU/L
C3 nephritic factor (C3NF)	Negative	Ratio > 1.022
C3	60	90–180 mg/dL
C4	13.3	10–40 mg/dL
RF	Negative	<15 IU/ml
ANA, anti-ds-DNA, ANCA, and cryoglobulin	Negative	NA
Anti-GBM	Negative	<1 AI
Anti-PLA2R Ab (ELISA)	Negative	NA
IgG	1060	800–1600 mg/dL
IgA	100	70–400 mg/dL
IgM	127	90–180 mg/dL
Urine protein	4.9	<0.03 g/gCr
Urine red blood cells	25–35	/HPF
Protein excretion	>300	mg/dl
SPEP M-protein concentration	3.83	Negative g/L
SIF	IgG *κ*	NA
Urine immunofixation electrophoresis: IgG *κ*	43.1	NA mg/dL
FLC *κ*	26.6	0.33–1.54 mg/dL
FLC *λ*	28.6	0.57–2.63 mg/dL
FLC *κ*/*λ*	0.93	0.26–1.65

AI: activity index, AU: arbitrary units, NA: not applicable, CFH: complement factor H, ANA: antinuclear antibody, anti-ds-DNA: anti-double-stranded DNA antibody, ANCA: antineutrophil cytoplasmic autoantibody, anti-GBM: antiglomerular basement membrane, RF: rheumatoid factor, anti-PLA2R Ab: antiphospholipase A2 receptor antibody, ELISA: enzyme-linked immunosorbent assay, SPEP: serum protein electrophoresis, SIF: serum immunofixation, FLC: free light chain, and HPF: high-power field.

## Data Availability

The data used to support the findings of this study are included within the article.
